# linkedISA: semantic representation of ISA-Tab experimental metadata

**DOI:** 10.1186/1471-2105-15-S14-S4

**Published:** 2014-11-27

**Authors:** Alejandra González-Beltrán, Eamonn Maguire, Susanna-Assunta Sansone, Philippe Rocca-Serra

**Affiliations:** 1 Oxford e-Research Centre, University of Oxford, Oxford, OX1 3QG, UK

## Abstract

**Background:**

Reporting and sharing experimental metadata- such as the experimental design, characteristics of the samples, and procedures applied, along with the analysis results, in a standardised manner ensures that datasets are comprehensible and, in principle, reproducible, comparable and reusable. Furthermore, sharing datasets in formats designed for consumption by humans and machines will also maximize their use. The Investigation/Study/Assay (ISA) open source metadata tracking framework facilitates standards-compliant *collection, curation, visualization, storage *and *sharing *of datasets, leveraging on other platforms to enable *analysis *and *publication*. The ISA software suite includes several components used in increasingly diverse set of life science and biomedical domains; it is underpinned by a general-purpose format, ISA-Tab, and conversions exist into formats required by public repositories. While ISA-Tab works well mainly as a human readable format, we have also implemented a linked data approach to semantically define the ISA-Tab syntax.

**Results:**

We present a semantic web representation of the ISA-Tab syntax that complements ISA-Tab's syntactic interoperability with semantic interoperability. We introduce the linkedISA conversion tool from ISA-Tab to the Resource Description Framework (RDF), supporting mappings from the ISA syntax to multiple community-defined, open ontologies and capitalising on user-provided ontology annotations in the experimental metadata. We describe insights of the implementation and how annotations can be expanded driven by the metadata. We applied the conversion tool as part of **Bio-GraphIIn**, a web-based application supporting integration of the semantically-rich experimental descriptions. Designed in a user-friendly manner, the Bio-GraphIIn interface hides most of the complexities to the users, exposing a familiar tabular view of the experimental description to allow seamless interaction with the RDF representation, and visualising descriptors to drive the query over the semantic representation of the experimental design. In addition, we defined queries over the linkedISA RDF representation and demonstrated its use over the linkedISA conversion of datasets from Nature' Scientific Data online publication.

**Conclusions:**

Our linked data approach has allowed us to: 1) make the ISA-Tab semantics explicit and machine-processable, 2) exploit the existing ontology-based annotations in the ISA-Tab experimental descriptions, 3) augment the ISA-Tab syntax with new descriptive elements, 4) visualise and query elements related to the experimental design. Reasoning over ISA-Tab metadata and associated data will facilitate data integration and knowledge discovery.

## Background

The movement for open science and open research data is being increasingly embraced by the research community, including researchers [1], funders and regulators [2,3], and journal editors [4,5]. Shared datasets are truly useful if the information is provided in a standardised manner to ensure they are comprehensible and, in principle, reproducible, comparable and reusable. Data management, sharing policies and plans have emerged to ensure the resulting data is well annotated and shared appropriately. The experimental context (or metadata) should be richly described following community-standards, where these exists, and the information should be made available in formats for both human and machine consumption. Several community-driven efforts aim to develop data reporting standards (e.g. [6-9]). Describing the experimental metadata, however, is a time-consuming task requiring user-friendly tools with two key features: implementation of standards in an *invisible *manner, hiding as much of their complexities as possible, and adaptability to the diverse types of studies and analytical techniques currently in use by researchers.

**Figure 1:**
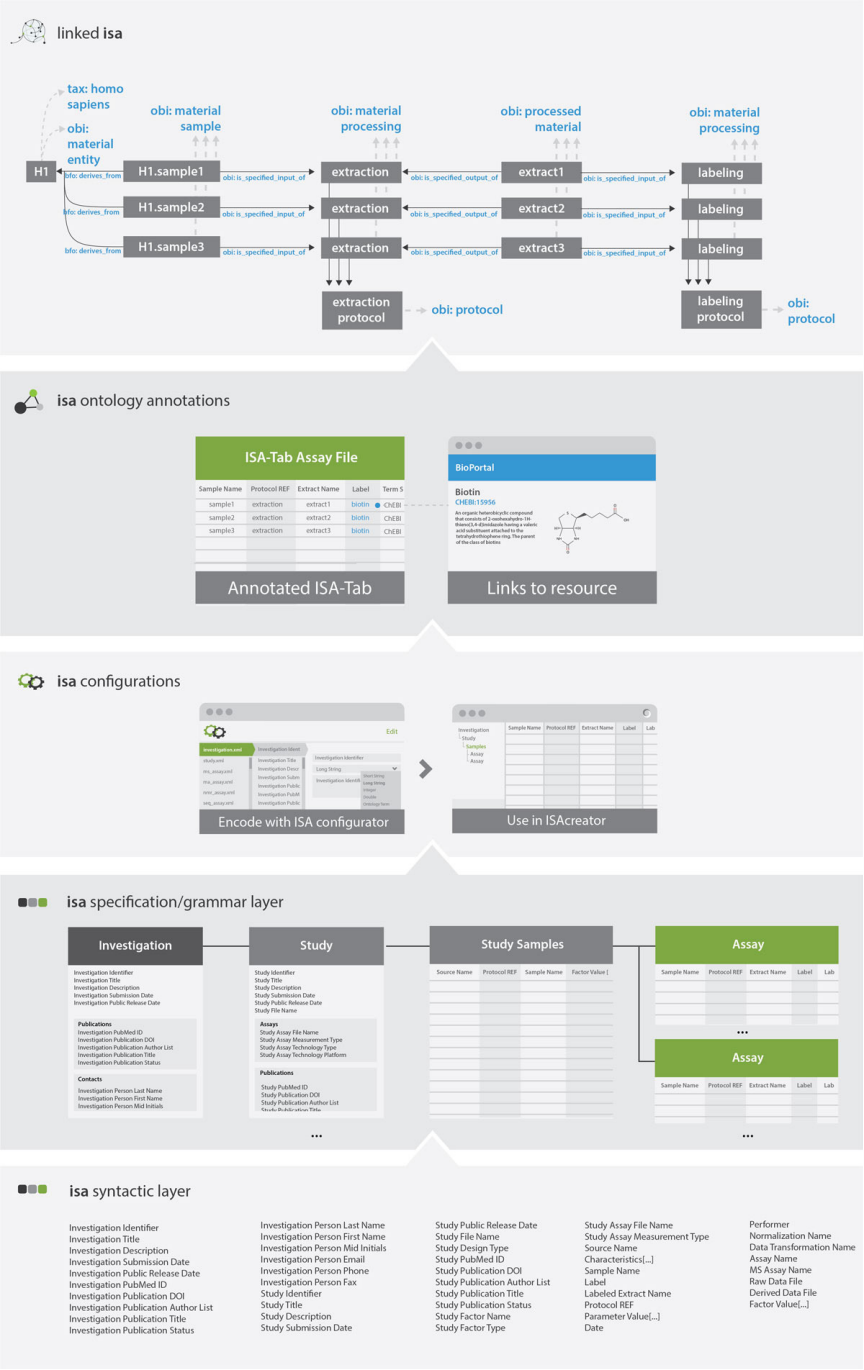
**ISA framework conceptual layers: from syntactic layer up to linkedISA layer**.

The Investigation/Study/Assay (ISA) metadata tracking framework [10] is an exemplar open source system that facilitates standards-compliant collection, curation, visualisation, storage and sharing of datasets. It was originally designed for multi-omics experiments in the life sciences but, since then, it has been applied to a variety of domains. It leverages other platforms to enable analysis and publication [11-13]. At the heart of the ISA framework, there is the general-purpose ISA-Tab file format [14], focusing on the description of the experimental metadata, and building on the *Investigation, Study *and *Assay *categories. The metadata in each of these categories is kept into three tab-delimited files, respectively. An *investigation *file maintains metadata about the project context and links to one or more *study *files. A *study *file describes a unit of research, describing the subjects of study and how they are obtained. Those subjects are then used in one or more *assay *files, which in turn, describe analytical measurements. The ISA-Tab format defines the syntactic elements of the ISA infrastructure and the ISA-Tab specification [15] determines the way in which the syntactic elements can be combined (see Figure 1, syntactic and specification layers).

The extensible, hierarchical structure of the ISA-Tab format enables the representation of studies employing one or a combination of assays and technologies, focusing on the description of its experimental metadata. Due to its general-purpose nature, the level of granularity of the descriptors and underlying semantics of the ISA-Tab format is customisable and depends on the type of experiment (e.g. a plant-based metabolomics study would require descriptors different from one studying gene expression in mammalian stem cells).

These customisations can be expressed as ISA configurations (or templates) for each specific type of assay, as defined by the entity they focus to measure and the technology they use (see Figure 1, configurations layer). Each template will define a specific arrangement of the ISA syntactic elements, *i.e*. a specific set of fields, to describe those assay types. For example, for a transcription profiling assay, *i.e*. the measurement is transcription profiling, using DNA microarray technology, the assay file structure starts identifying the *samples*, which are transformed into an *extract *and then a *labeled extract*, where each transformation is defined as an application of a *protocol*. The *protocol *itself is defined in the *investigation *file and referenced from the *assay *file.

The tabular format was chosen because it is user-friendly, as most scientists are familiar with spreadsheet manipulation software. While the format provides syntactic interoperability, the underlying semantics of the tabular format are left to the users interpretation and it is not amenable for straightforward machine manipulation and elucidation.

The ISA-Tab specification encourages users to provide ontology annotations, aiming at assigning unambiguous definitions of elements and harmonisation across ISA-Tab datasets (see Figure 1, ontology annotations layer).

Accompanying the format, the ISA software suite [11,14,16,17] allows to create and edit ISA-Tab files but also to persist, store, serve and convert them to a growing number of related formats. The framework is used by an expanding community, the ISA Commons [18] to deliver a growing ecosystem of public and internal resources - in increasingly diverse set of life science and biomedical domains -- ranging from *international public repositories *[19], *institutional repositories *[20] to *funded research consortia *[21] and data journals [12,13].

Semantic web standards [22] offer a variety World Wide Web Consortium (W3C) recommended technologies to support data sharing and reuse in a machine readable format, susceptible to automatic inferencing. These technologies enable information integration and re-use of common vocabularies. Among them, the Resource Description Framework (RDF) [23] is a standard model for data exchange and integration on the web. RDF represents information in the form of statements, which take the form of triples *<subject, predicate, object>*. The *subject *refers to the resource being described, the *predicate *is the property or relation being considered, and the *object *is another resource or a specific value, representing the value assumed by the subject for the specific property. The *subject *and *predicate *are identified with Uniform Resource Identifiers (URIs), and the *object *can be a URI or a literal value. URIs identify the names of resources and are a key component of the semantic web standards. They allow for global naming and referencing to web resources.

On top of the RDF data representation, a common domain model is required for applications to be able to exchange meaningful information. This is achieved with a vocabulary layer composed of standards such as the RDF Schema (RDFS) [24] and the Web Ontology Language (OWL) [25]. RDFS offers ways to define class/property hierarchies and domain/range of properties. OWL extends RDFS, offering more expressivity (e.g. inverse, transitive, symmetric, functional or inverse functional properties, cardinality restrictions on classes) tied in a logics-based formal framework relying on description logics [26].

In this work, we introduce a novel methodology to transform the ISA-Tab format (ISA-Tab) into RDF (see Figure 1, linkedISA layer). We introduce a new software component of the ISA framework: the linkedISA conversion tool, relying on mappings from the ISA syntax to multiple community-defined, open ontologies. We present an evaluation of the resulting RDF representation by running queries over it, and showing that the ISA-Tab information is preserved and extended with newly added entities. The queries were applied in **Bio-GraphIIn **[17], a web-based application supporting integration of the semantically-rich experimental descriptions. We also demonstrate the queries over the conversion of the Nature Scientific Data journal ISA-Tab datasets [13].

This transformation to RDF complements ISA-Tab syntactic interoperability with semantic interoperability and exploits the ontology annotations available in the ISA-Tab format. The conversion relies on mapping files to specify the semantic framework to be used, allowing the users to convert ISA-Tab files into semantic representations relying on different OWL ontologies. The canonical mapping provided within the linkedISA tool adopts the Ontology for Biomedical Investigation (OBI) [27] and core ontologies under the Open Biological and Biomedical Ontologies (OBO) Foundry umbrella [8]. But, mappings to the provenance ontology (PROV-O) [28] and to the SemanticScience Integrated Ontology (SIO) [29] are also included.

## Implementation

### The linkedISA converter engine

As presented in the introduction, ISA-Tab is a tabular or spreadsheet-like format designed to describe biological experiments using a combination of technologies.

**Figure 2:**
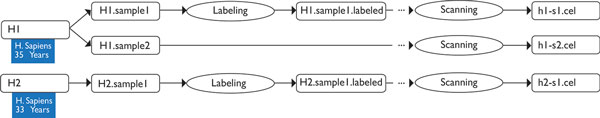
**Graph representation of an ISA-Tab file**.

The underlying model of the ISA-Tab format is a direct acyclic graph with nodes representing material entities or data, and edges representing transformation between nodes (see Figure 2), namely:

1. processes acting on material entities (such as an organism) and yielding other material entities

2. processes acting on material entities and yielding data

3. processes acting on data and yielding data

**Figure 3:**
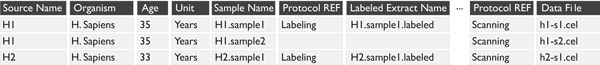
**ISA-Tab example**.

The ISA graph representation is kept as a table (see Figure 3), to enable manipulation using spreadsheets-based software.

The format and software tools to create ISA-based experimental descriptions (e.g. ISAcreator [14] and OntoMaton [16]) support assigning specific semantics to a number of syntactic elements. So, for example, the source biological material in the experiment can be specified to be from a human and annotated with ontology terms. In the ISA-Tab syntax, this will mean associating a *Characteristics[] *(or attribute) to the *Source Name *material in the *study *file, which describes how the samples are derived from source material. This is achieved by juxtaposing the column *Source Name *and a column *Characteristics[OBI:organism]*, where the text in between the square brackets is specific for this study and means that the source attribute is its organism. This attribute is identified by the OBI term whose URI is http://purl.obolibrary.org/obo/OBI_0100026. In a specific row describing one of the biological sources, the annotation could be [NCBItaxon:Homo sapiens], indicating that the source was a human using the URI http://purl.obolibrary.org/obo/NCBITaxon_9606 from the National Center for Biotechnology Information (NCBI) taxonomy [30].

**Figure 4:**
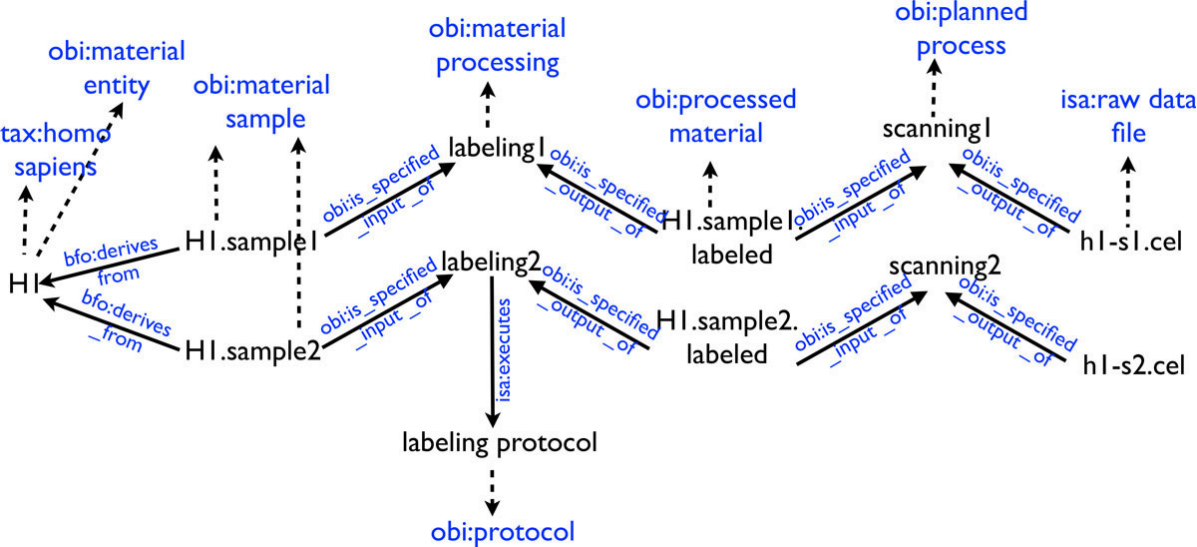
**RDF representation example**.

The linkedISA conversion exploits knowledge about the ISA-Tab specification to obtain a semantically-rich interpretation of ISA-Tab into RDF (see Figure 4). When available, the transformation exploits the ontological annotations present in ISA-Tab.

As the conversion needs to cater for a broad user community (see ISA Commons [18,31]), we decoupled the conversion engine from a specific semantic framework, *i.e*. a set of ontologies relevant for a specific group or resources. Users can configure the tool to include one or more mapping files, which associate ontological entities to the ISA-Tab syntax elements. The mapping files are described in more detail in the section entitled **linkedISA semantic framework**.

We will now demonstrate some of the RDF representation choices, and how they encapsulate the ISA-Tab syntactic elements and not only explicit relationships between the elements, but also relationships that are implicit in ISA-Tab. Since its inception, the ISA-Tab format has been applied to a variety of datasets and domains, and it has proven to be robust allowing for the description of the experimental steps. In this work, we take advantage of the flexibility and extensibility of semantic web models to incorporate further information derived from ISA-Tab. Adding this information back in ISA format is not necessary, as it can be derived, but is provided in the linked ISA representation for improved availability.

**Figure 5:**
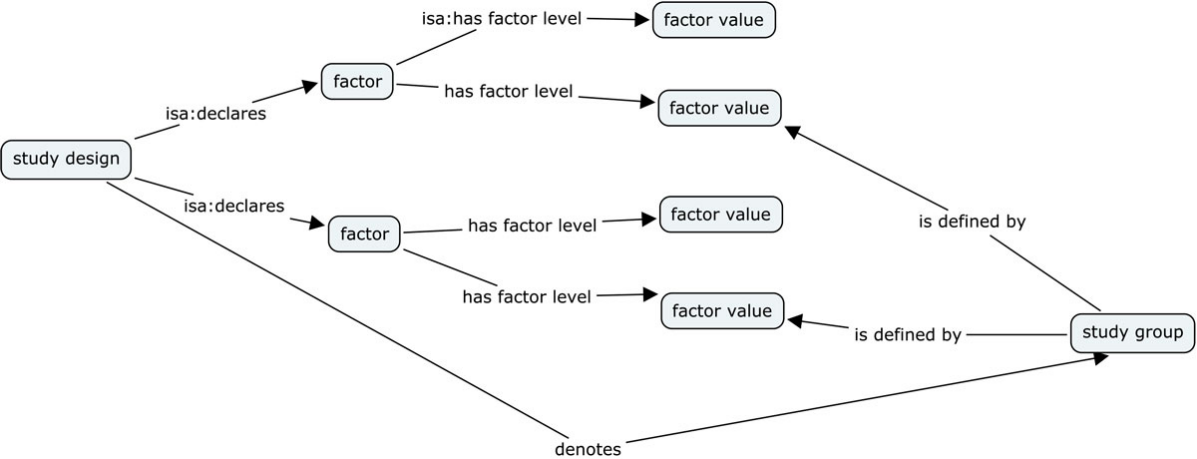
**Representation of ISA factors**.

We will show how *study design *and *study factors* (from ISA-Tab) are represented and how *study groups *(not available in ISA-Tab explicitly) are represented. The *study design *and the *study factors *-- the independent variables of a study -- are declared in the *investigation *file, indicating their name and their type and can be annotated with ontology terms. Figure 5 shows the RDF representation of the relationship between *study design, factors*, their *values *and *study groups*.

In ISA-Tab, the *study sample *and *assay *tables may then refer to the declared factors and assign a vector of *Factor Value*s for each of the samples (in each row of the tables), thereby defining an implicit group membership. Factor values can be present in both, study and assay files. For experiments looking into tuning data acquisition techniques, as often the case in comparison between methods, the independent variable levels do not affect the samples. Therefore, the factor values only make sense at the assay level. On the other hand, when the factor values affect biosamples, as it is usual in intervention studies, they should appear in both files. Given this uncertainty, we chose not to include an explicit relationship between samples and factor values in RDF (see Figure 5), as it is the more generic case. However, for specific datasets or domains, our mapping could be extended to relate *study factors *and *samples*.

Irrespective of this issue, the linkedISA conversion enriches the ISA-Tab representation by adding entities not explicitly available in the ISA syntax thanks to an interpretative layer relying on the analysis of the experimental graph. *Study groups*, defined by the combination of factor values, are automatically added to the graph as well as their respective sizes, corresponding to the count of their members.

In the next section, we will show how the semantic representation enables data-driven annotation through queries allowing to produce new information from the relationships established in RDF, going beyond the ISA-Tab grammar. To demonstrate this ability, we will rely on examples related to study factors and study groups, as described above.

### Queries over the linkedISA representation

The SPARQL Protocol And RDF Query Language (SPARQL) is the recommended query language for RDF data in the Web or in a triple store [32]. In this section, using a set of demonstrative SPARQL queries, we highlight the benefits of our linkedISA RDF representation, by retrieving essential information about study design, otherwise not easily recoverable from ISA-Tab, or similar experimental representations [33]. In this section, we present several SPARQL queries that can be ran across multiple ISA-Tab studies converted to RDF using the linkedISA package.

The first SPARQL query considered, available as *Additional File*1 is enough to retrieve in one go a study factor and its associated discrete levels for each study. This is a major improvement resulting from the RDF conversion, which makes relationships between factors and their values explicit (see Figure 5). The equivalent query over the ISA-Tab representation requires inspecting the whole *study *or *assay *tables to pick up the values specified in the *Factor Values *columns. Furthermore, the SPARQL query at *Additional File*2 is an expansion of *Additional File*1 to count the number of levels per factor for each study.

Exploring further the overall structure of the experiment, the SPARQL query at the *Additional File*3 enables direct access to important information such as the nature and sizes of the different study groups, defined as the combination of factor values. While the ISAcreator tool offered functionality to visualise the various study groups, up until now, there was no simple way to readily interrogate the groups from ISA-Tab.

Having information about the study groups and their sizes, it is possible to determine if the experiment follows a balanced or imbalanced design. A set of queries, ran in a stepwise fashion, evaluates the nature of the design, and adds the relevant triple to the knowledge base indicating that newly discovered fact. In so doing, the RDF representation enables automatic annotation of key characteristics of the experiment. This procedure also enables to detect experiments that might have been wrongly annotated (e.g. an imbalanced design that had been annotated as balanced). This example illustrates one of the many opportunities for simplifying and improving the curation workflow for the end user.

The query in *Additional File*4 retrieves the minimum and maximum group sizes for each study. The query at *Additional File*5 answers the question *'is the study design balanced?' *by checking if the minimum value is equal to the maximum value. Finally, we use SPARQL CONSTRUCT [32] to add a new triple relying on the *balanced design *term defined in the STATistics Ontology (STATO) [34]. The SPARQL CONSTRUCT query can be found at *Additional File*6.

The queries presented here demonstrate the importance of good curation and annotation practices, where experimental descriptions are harmonised across datasets. This linkedISA representation exploits the harmonisation and enables to explore the data and support linking across datasets.

Furthermore, understanding the overall study design provides an insight into possible comparisons or contrasts with other study groups and/or experiments. The design information can also be used to validate the experiment analysis results, which should be available as data matrices or tables. Last but not least, these matrices of analysis results may have annotations, for example using the STATO ontology [34], whose consistency with the experimental annotations will be amenable to automatic validation.

### linkedISA semantic framework

An important goal for our project's semantic web development is to provide a flexible approach capable of accommodating the needs of the various communities using the ISA-Tab format for data management and data publication. This translated into the delivery of software allowing distinct semantic frameworks -- preferred ontologies used by given communities -- to be considered for the conversion to RDF. Various mappings are therefore proposed between relevant ontologies and the ISA-Tab syntax, and implicit entities are deduced from ISA-Tab graph inspection. This architectural choice provides adequate levels of customisation while retaining a single conversion core engine.

The format of the mapping files is tabular with the following pattern:

$$\overset{\text{type definition for ISA syntactic element}}{\overset{︷}{≺\mathrm{ISA}\text{ syntactic element}≻≺\text{ontology class label}≻≺\text{ontology class URI}≻}}$$

$$\overset{\text{one}\;\text{or}\;\text{more}\;\text{property}\;\text{relations}\;≺\text{predicate}≻≺\text{object}≻}{\overset{︷}{≺\text{ontology}\;\text{property label}≻≺\text{ontology}\;\text{property}\;\text{URI}≻≺\text{ontology}\;\text{class}\;\text{label}≻≺\text{ontology}\;\text{class}\;\text{URI}≻\cdots }}$$

The default mapping files offered with the linkedISA tool condense the semantic interpretation of the ISA-Tab syntax, which together with the business logic embedded in the conversion process, produce an RDF representation that is generic and caters for the different domains in which ISA-Tab is used. Being generic means that the mappings do not cover user-defined annotations allowed by the ISA-Tab syntax, such as the type for the characteristics or parameters (*i.e. Characteristics[ xxx ] *and *Parameter Value[ xxx ] *), and these are associated to their bearers (samples and processes, respectively) with generic properties. While we have captured some of this knowledge in linkedISA (*e.g*. the presence of *Characteristics[OBI:organism]* is used to set the type for the sample), it is less than trivial to support the endless options ahead of time. However, specificity can be achieved by supplying linkedISA with new mapping files to replace or augment the generic transformation. For instance, where the ISA study sample file contains the following pattern:

Source Name    Characteristics[OBI:organism]    Characteristics[OBI:tissue specimen] 

a new mapping file could contain an specific relationship between the two *Characteristics *indicating that the tissue is part of the organism:

Characteristics[OBI:tissue specimen],tissue,http://purl.obolibrary.org/obo/OBI_0001479, is part of, http://purl.obolibrary.org/obo/BFO_0000050, organism, Characteristics[OBI:organism]

**Figure 6:**
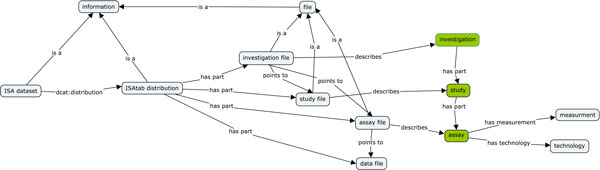
**Example ISA terminology**.

In order to define the specific semantics of the ISA representation, and to work as a backbone for the multiple mappings, we defined the ISA ontology [35]. This ontology (see Figure 6) formally defines the ISA terminology and maps, when relevant, to existing ontologies and re-using their terms.

The mapping files were generated using OntoMaton widget for Google Spreadsheets [16], as it allows to search and store the URIs for the relevant terms, while restricting the search over specific ontologies. The mapping files are available in the GitHub repository together with the linkedISA code.

**ISA-ISA mapping: **This mapping is a one-to-one relationship from the ISA syntax to the ISA ontology.

**ISA-OBI mapping **The ISA-OBI mapping relates ISA concepts to OBI concepts. In some cases the mapping is straightforward (e.g. ISA *Study Assay *corresponds to *OBI:assay *(http://purl.obolibrary.org/obo/OBI_0000070), but in other cases, the mapping encapsulates ISA knowledge that might not be immediately transparent by inspecting the ISA syntax.

For example, let's consider *Protocol REF *elements, which always refer to the application of a *Protocol *defined in the *Investigation *file, and might appear both in *Study *or *Assay *files. The *Study *file describes the samples and the application of *Protocol REF *refers to *OBI:material processing *(http://purl.obolibrary.org/obo/OBI_0000094). On the other hand, in the *Assay *file, a *Protocol REF *refers to the more generic term *OBI:planned process *(http://purl.obolibrary.org/obo/OBI_0000011) as it can be either a process converting material into more material, material into data, or a data transformation.

**ISA-SIO mapping: **The SIO ontology has been used for the annotation of the resources available in BIO2RDF [36]. Thus, in order to enable interoperability of ISA datasets with those resources, we provide a mapping between the ISA syntax and SIO.

**ISA-PROV-O mapping: **Provenance information refers to the relationship between entities, activities and agents (e.g. people) involved in producing new entities (e.g. data or material). A W3C working group dedicated to generating a model and multiple serializations for representing provenance information on the web, produced the provenance ontology (PROV-O) [28]. While similar information is covered in ontologies such as OBI, the converter accepts mapping between the ISA syntax and PROV-O in order to supply a straightforward way of interoperating with resources relying on this model.

### linkedISA identifiers

Identity methods for each element of the ISA syntax, *i.e*. ways to determine the uniqueness of an element, are very important considerations for the linkedISA conversion. While some entities in the ISA-Tab representation are uniquely identified by the strings that represent them (*e.g*. material entities such as *Source Name *and *Sample Name*), other entities that appear several times in the ISA-Tab representation, follow different rules. For example, *Protocol REF *elements are distinguished depending on the combination of their name, parameters, inputs and outputs. Hence, while two rows corresponding to a *Protocol REF *in an *Assay *file may have the same name, they may be, in fact, referring to a different application of the same protocol.

Consequently, linkedISA creates identifiers, URIs, for each particular element in an ISA dataset by embedding ISA knowledge to determine when the element is new or has already been used in the data description.

Additionally, the converter allows users to specify a <base IRI> for the generation of identifiers, and then follows the pattern:

<base IRI> / <type> / <counter>

where <type>refers to the type of the individual and <counter>enumerates the individuals of a type in order of creation.

For example, the individual representing the soapdenovo2 ISA dataset [37], whose base IRI is defined as

http://w3id.org/isa/soapdenovo2/

will have the following IRI:

http://w3id.org/isa/soapdenovo2/isa_dataset/1

### linkedISA architecture

**Figure 7:**
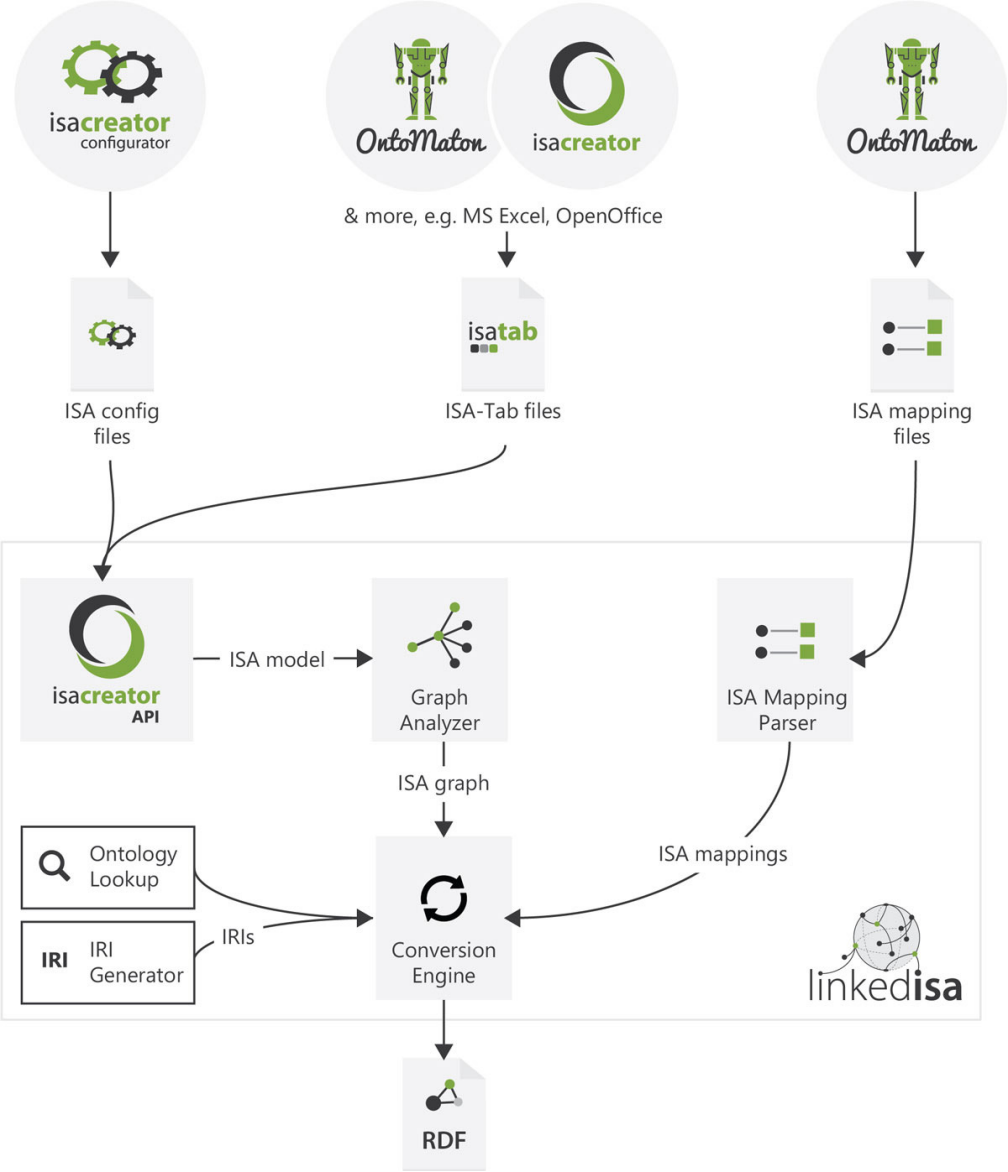
**linkedISA high level software architecture**.

Figure 7 presents linkedISA high-level software architecture. We describe briefly each of the components:

**ISAcreator API: **it is used to parse ISA configuration and ISA-Tab files.

**Graph analyser module: **a module to identify the direct acyclic graph underlying the tabular representation, it determines the different node types and keeps information required for the RDF conversion.

**ISA mapping parser: **a module in charge of parsing the ISA mapping files, which associates the ISA syntax elements to different ontological representations. This module identifies both: mappings corresponding to types for the ISA elements and property mappings. In some cases, mappings are maintained per type of element (e.g. all the mappings corresponding to *Study Group*s, to facilitate conversion to RDF for a subset of the instances generated).

**linkedISA converter: **this is the module in charge of conversion to RDF by relying on the mappings information. It encapsulates the logic about identity methods for each ISA element and relies on **Ontology Lookup **and **IRI generator **functionality to retrieve or to create IRIs for ontology annotations.

The implementation of linkedISA was perfomed in the Java language and it relies on the OWLAPI Java API [38].

### linkedISA assessment

In order to assess the linkedISA conversion tool, from ISA-Tab to RDF relying on mappings from the syntax to relevant ontologies, we converted multiple ISA-Tab datasets, devised and applied numerous SPARQL queries. The main aim of the queries is to demonstrate that the RDF representation can retrieve all the information available in ISA-Tab. Also, the information provided by previous interfaces in the ISA framework (such as the BioInvestigation Index [14] storage solution). But also, the new representation can retrieve additional information that was not possible to retrieve before.

In the following subsections, we describe several use cases where the linkedISA conversion was applied:

• the Bio-GraphIIn web application [17]

• queries over Nature's Scientific Data ISA-Tab datasets, available in a SPARQL endpoint [39]

• a reproducibility case study, where the metadata was represented in ISA-Tab and the linkedISA conversion was applied [37]

• an application of linkedISA conversion in the biodiversity domain [40]

### Bio-GraphIIn: an linkedISA-backed web application

Public repositories of subject based experiments and trials (e.g. [19,41]) are remarkable resources, allowing access to thousands of datasets for exploitation and mining. However, a closer inspection reveals that few of these repositories are geared to make it easy to query, slice and dice datasets. In fact, assembling a meta-dataset means running keyword-based queries to identify relevant individual studies, downloading those and processing them locally to filter and sub-select samples and data files of interest meeting a number of inclusion criteria. This points to current limitations in the way to access and query those major resources. First of all, it is not possible to explore datasets by interrogating some of their design features. It is currently impossible to filter experiments with a minimum of 3 replicates per treatment groups or obtaining only studies with a balanced design. Then, downloading groups of samples and associated information instead of the entire datasets is seldom, if ever, possible.

With these issues in mind, we explored the requirements and implementation of a novel storage and visualization solution for the ISA infrastructure: the Biological Graph Investigation Index or Bio-GraphIIn (pronounced *bio-graphene*).

**Figure 8:**
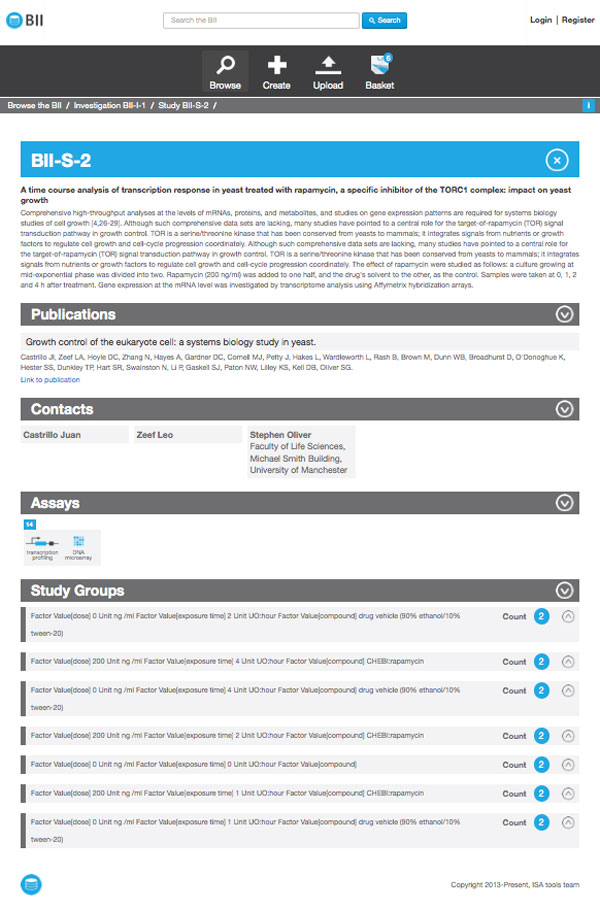
**Bio-GraphIIn interface showing study groups**.

**Figure 9:**
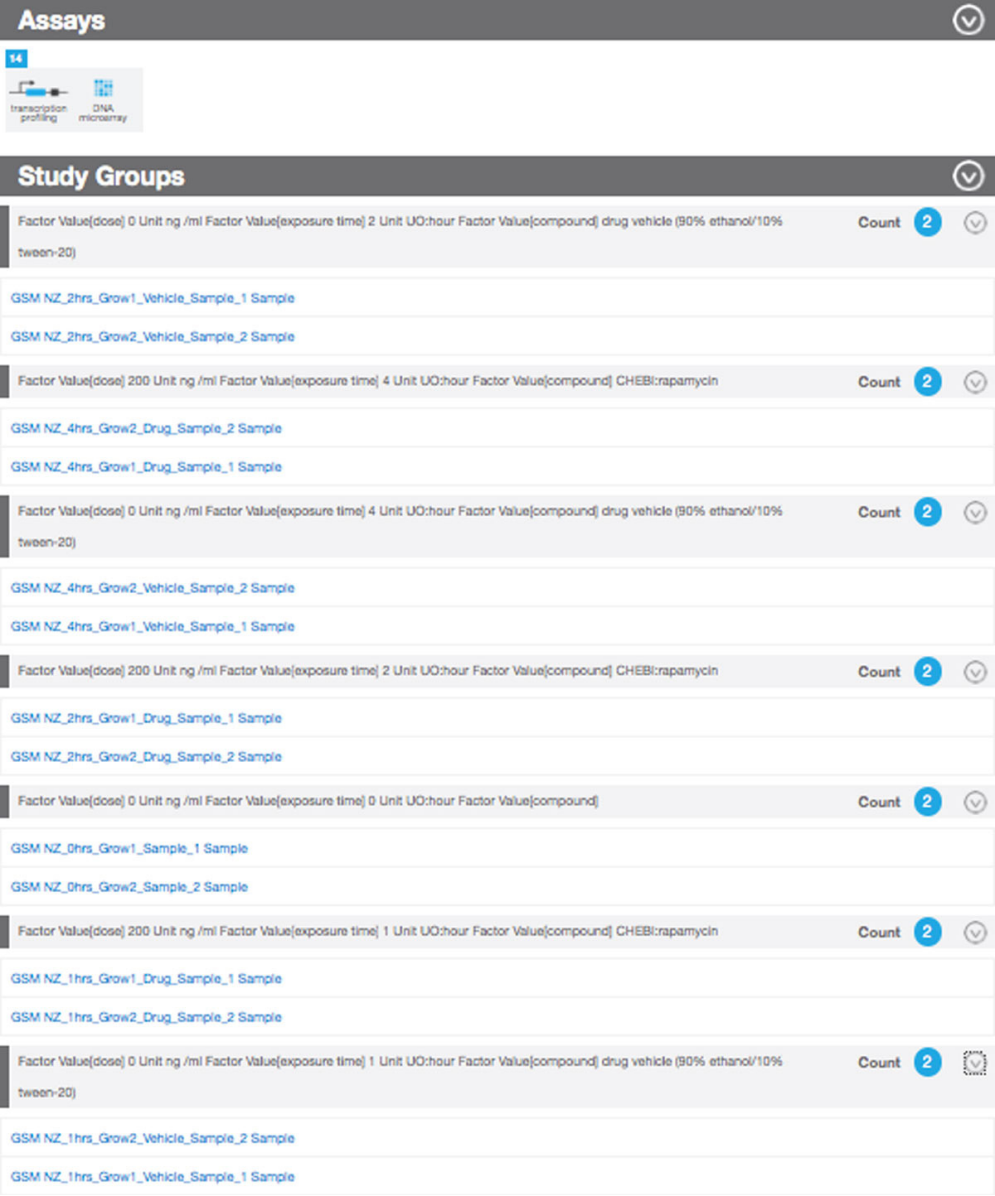
**Bio-GraphIIn interface showing study group members**.

Table 1 shows the functionality provided by existing repositories of *omics *data against the requirements for Bio-GraphIIn. Some of these requirements were tackled in a prototype implementation that depends on the linkedISA conversion, as described in [17]. As a result, SPARQL queries were written to retrieve all the information shown in Bio-GraphIIn interface (See Figures 8 and 9 for examples of Bio-GraphIIn graphical user interface). The use of the RDF representation in Bio-GraphIIn has facilitated answering queries such as those described in the section.

**Table 1:** Repositories functionality and Bio-GraphIIn requirements.

	Data Types	Format	Browsing/ Searching	Programmatic submission	Programmatic access	CRUD operations	Community curation	RDF
**BioSample DB**	sample info	Sample- TAB	browse/search	X (email submission)	REST API	X	X	YES

**ArrayExpress/GEO**	Sequencing	MAGE- TAB	browse/filter/ search/ advanced search	MAGE-TAB spreadsheet / MIAMExpress	REST API	X	X	X*

**SRA/ENA**	next generation sequencing	SRA-XML	browse/text/ sequence/ advance search	Webin, REST	REST API	X	X	X

**PRIDE**	mass spectrometry	PRIDE-ML	PRIDE inspector/ PRIDE Biomart	X (FTP upload)	Java API	X	X	X

**BII**	All	ISA-TAB	browse/text search/filtering	X	SOAP web services	X	X	X

	

**Bio-GraphIIn**	All	ISA-TAB	browse/filter/ search/ advanced search	YES (upload, REST)	REST API	YES	YES	YES

^*^We are referring to the ArrayExpress repository not to the Expression Atlas, which is available in RDF

### Nature's Scientific Data ISA-Tab datasets

The ISA-Tab format has been adopted by the recently launched new data publication from Nature Publishing Group: Scientific Data [42]. Scientific Data publishes formal descriptions of datasets in the form of Data Descriptors, which are accompanied with structured metadata in ISA-Tab, following a specific Scientific Data configuration. The currently available data descriptors range over a variety of domains, including neuroscience, stem cells research, metabolomics, transcriptomics.

To demonstrate the linkedISA conversion, we transformed all currently available Scientific Data ISA-Tab datasets and loaded them into a Virtuoso instance, within a named graph (http://w3id.org/isa/sdata). Over this instance, we ran all the SPARQL queries presented before. For more information about the SPARQL endpoint and to run the queries over the linkedISA representation, see the linkedISA website:

http://isa-tools.github.io/linkedISA/

### Reproduciblity study

The linkedISA tool was also applied SOAPdenovod2 case study [37]. This was an analysis of the reproducibility of the SOAPdenovo2 genome assembler results presented in the GigaScience journal, where Galaxy workflows were produced and combined with different data models for the description of the experimental steps (ISA-Tab), the preservation of the workflows (Research Objects) and the representation of the main results (nano publications). Several SPARQL queries were produced and ran over the linkedISA information and details can be found in the SOAPdenovo2 case study website [37].

### Biodiversity study

In [40], the linkedISA conversion was applied in the context of environmental sciences. A modular modelling approach was followed where: an ISA-Tab template was generated for collecting data of a biodiversity assay and then, a semantic representation of the templates was devised, extending the canonical linkedISA conversion with domain-specific elements such as sequencing techniques applied in biodiversity context.

## Discussion

A number of projects have applied semantic web technologies to the description of life science experimental data.

Wang et al [43] discussed the advantages of using RDF over the eXtended Markup Language (XML) for representing and integrating *omics *data. They emphasized the syntactic and document-centric nature of XML, preventing to reach the level of interoperability required by current dynamic bioinformatics systems.

McCusker *et al *[44] worked on representing the provenance of microarray experiments by converting the MicroArray Gene Expression Tabular (MAGE-Tab) format [45] into RDF. Mapping MAGE files into the Open Provenance Model (OPM) and the Proof Markup Language (PML), they demonstrated the feasibility by converting one experiment from the ArrayExpress database [46] as a step towards a vision of a uniform representation of provenance in a translational research pipeline. As our work is based on the ISA-Tab format, which is used to represent multi-omic (and multi-assay) experiments and expands beyond MAGE-Tab envelop, our conversion tool encompasses McCusker *et al *results. We currently provide a mapping to the PROV-O ontology as default. Other provenance models can be added, simply by providing an additional mapping file to our converter, a feature which illustrates the flexibility of our implementation, as it decouples the mapping from the conversion engine.

Deus et al [47] also presented a methodology using RDF for integrating microarray-based transcriptomics experimental descriptions from three different source representations: the Gene Expression Atlas [48], the W3C BioRDF task force [36] and the Harvard Stem Cell Institute (HSCI) blood genomics project [49]. Each of the repositories had an independent RDF conversion of the microarray-based data, relying on different ontologies. Deus *et al *identified a set of patterns to transform SPARQL queries to interrogate the three diverse RDF representations of the distributed microarray experiments resources. Their work focused on reporting gene expression data only.

Anguita *et al *[50] also worked in providing an RDF interface to data sources based on MAGE standards. Their tool, called RDFBuilder, acts as a wrapper service supporting SPARQL queries over ArrayExpress data. They do not rely on external ontologies to convert the microarray data. Instead, RDFBuilder converts the MAGE Markup Language (MAGE-ML), *i.e*. the database schema, into RDF, rather than the actual data.

The ToxBank project [21] has provided a transformation from ISA-Tab to RDF with their isa2rdf code [51], which relies on Jena RDF API [52] and ToxBank specific vocabularies. While similar in scope, the isa2rdf is specialized and does not provide the OWL support we sought.

## Conclusions

The Investigation/Study/Assay (ISA) metadata tracking framework -- composed of the ISA-Tab format and multiple open source software tools -- facilitates standards-compliant *collection, curation, visualization, storage *and *sharing *of datasets, leveraging on other platforms to enable *data analysis *and *data publication*.

This manuscript introduced the linkedISA conversion software that transforms ISA-Tab formatted experimental information into RDF triples, following the linked data approach. The conversion relies on a mapping file, resolving the ISA syntax into popular BFO-based ontologies. When available, the linkedISA representation capitalizes on the existing ISA-Tab ontology-based annotations provided by the user. Furthermore, exploiting the extensibility characteristics of RDF, it augments annotation with new elements derived from what implicit information stated in ISA-Tab. ISA-Tab syntactic interoperability has been complemented by semantic interoperability, by producing a machine processable and semantically rich representation of ISA-Tab formated data.

We underlined the generic nature of the conversion and provided a roadmap to deal with domain specific complex representation in ISA-Tab format. Indeed, the conversion can be refined via new mapping files to replace or augment the generic mappings we provide, so that the specific patterns can be processed with the necessary level of detail and accuracy.

We demonstrated some of the advantages of the generated linkedISA representation by:

1. presenting a set of queries that illustrates how the linkedISA representation enables easy retrieval of experimental information, not available otherwise.

2. showing how SPARQL CONSTRUCT statements can be harnessed to produce automatic annotations of experimental metadata. The procedure allows to perform metadata-driven annotation automatically, without human intervention. Quality checks of existing annotations (e.g. identifying that an experiment had been incorrectly tagged) or generation of new annotations can therefore be carried out based on curator-defined rules or information on ontological artefacts.

3. presenting several examples where the linkedISA conversion has been applied: the Bio-GraphIIn web application (that provides new ways to explore and query datasets, clearly showing the different study groups and allowing cohort creation across studies), conversion of Nature's Scientific Data ISA-Tab datasets, a reproducibility case study over a GigaScience's article and a pattern for biodiversity datasets.

Ongoing work covers the following plans:

• explore more applications of the linkedISA conversion, where the RDF representation is capitalised to compare datasets and to slice and dice datasets according to their attributes

• apply the linkedISA tool to domain-specific ISA-Tab datasets, such as the metabolomics data available in the EBI Metabolights database [19,53]

• consider further domain-specific patterns for customising ISA conversion. Initial work done for microbial communities and biodiversity assays [40] is being evaluated.

• expand the conversion tool beyond the current experimental steps represented in ISA-Tab, to consider analysis results, by relying on the STATistics Ontology (STATO) [34]

Finally, we would like to underline how this work highlighted the value of good curation and annotation practices as their implementation and enforcement significantly impact the meaningfulness of the conversion and the downstream possibilities of data exploration and linking. As more experience is gained, we hope to further document best practices.

## Availability

**Project name: **linkedISA

**linkedISA website: **http://isa-tools.github.io/linkedISA/

**linkedISA converter engine: **https://github.com/ISA-tools/linkedISA

**Operating system: **platform independent

**Programming language: **Java

**License: **CPAL

**linkedISA RESTful service: **https://github.com/ISA-tools/linkedISA-ws

**Bio-GraphIIn web application: **http://bii.oerc.ox.ac.uk/

## Supplementary Material

Additional file 1 **Retrieve factors and their levels**. Click here for file

Additional file 2 **Count factor levels per factor**. Click here for file

Additional file 3 **Count number of members per study group**. Click here for file

Additional file 4 **Retrieve the min and max group sizes**. Click here for file

Additional file 5 **Is it a balanced design?**. Click here for file

Additional file 6 **Construct triple for balanced design**. Click here for file

## Declarations

The publication costs for this work were funded by EU COSMOS EC312941.

This article has been published as part of *BMC Bioinformatics *Volume 15 Supplement 14, 2014: Semantic, social, mobile applications for bioinformatics and biomedical laboratories: selected works from the 13th International Workshop on Network Tools and Applications in Biology (NETTAB 2013). The full contents of the supplement are available online at http://www.biomedcentral.com/bmcbioinformatics/supplements/15/S14.
